# A new threshold selection method for species distribution models with presence‐only data: Extracting the mutation point of the P/E curve by threshold regression

**DOI:** 10.1002/ece3.11208

**Published:** 2024-04-01

**Authors:** Boyang Yu, Wenyu Dai, Siqing Li, Zhaoning Wu, Jiechen Wang

**Affiliations:** ^1^ Jiangsu Provincial Key Laboratory of Geographic Information Science and Technology, Key Laboratory for Land Satellite Remote Sensing Applications of Ministry of Natural Resources, School of Geography and Ocean Science Nanjing University Nanjing China; ^2^ Jiangsu Center for Collaborative Innovation in Geographical Information Resource Development and Application Nanjing China

**Keywords:** predicted‐to‐expected curve, presence‐only, species distribution model, threshold, threshold regression

## Abstract

Selecting thresholds to convert continuous predictions of species distribution models proves critical for many real‐world applications and model assessments. Prevalent threshold selection methods for presence‐only data require unproven pseudo‐absence data or subjective researchers' decisions. This study proposes a new method, Boyce‐Threshold Quantile Regression (BTQR), to determine thresholds objectively without pseudo‐absence data. We summarize that the mutation point is a typical shape feature of the predicted‐to‐expected (P/E) curve after reviewing relevant articles. Analysis based on source‐sink theory suggests that this mutation point may represent a transition in habitat types and serve as an appropriate threshold. Threshold regression is introduced to accurately locate the mutation point. To validate the effectiveness of BTQR, we used four virtual species of varying prevalence and a real species with reliable distribution data. Six different species distribution models were employed to generate continuous suitability predictions. BTQR and nine other traditional methods transformed these continuous outputs into binary results. Comparative experiments show that BTQR has advantages in terms of accuracy, applicability, and consistency over the existing methods.

## Graphical abstract

This study proposes a new method named Boyce‐Threshold Quantile Regression (BTQR). The method uses threshold regression to locate the mutation point of Boyce's predicted‐to‐expected curve. Analysis based on source‐sink theory suggests that this mutation point may represent a transition in habitat types and serve as an appropriate threshold. Comparative research shows that BTQR has significant advantages in accuracy and stability over prevalent methods.

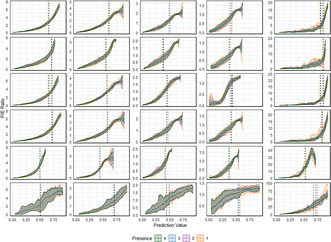

## 1 INTRODUCTION

Species distribution models are numerical tools that combine species distribution data with environmental data to estimate the species' occurrence probability, habitat suitability, and species richness (Elith & Leathwick, 2009; Guisan & Thuiller, 2005), which are widely used to provide information for ecological and biogeographic theories and conservation decisions (Franklin, 2013; Newbold, 2010). Many species distribution models generate continuous prediction results. Although some studies suggest that continuous results retain more information (Araújo et al., 2002; Vaughan & Ormerod, 2005), threshold classification approaches that convert continuous original predictions into binary outputs have been widely used in many ecological studies. Researchers employ thresholds for two major reasons: First, applied issues such as biodiversity evaluation (Pinto‐Ledezma & Cavender‐Bares, 2021), conservation planning (Bellamy et al., 2013), and climate change effect assessment (Bayliss et al., 2022; Charney et al., 2021; Della Rocca et al., 2019; Della Rocca & Milanesi, 2020, 2022a; Howard et al., 2023; Mori et al., 2018) generally require explicit binary discrete predictions of “presence/non‐existence” or “habitat/non‐habitat.” Second, the binary output facilitates the estimation of species‐related information, such as species prevalence.

The approaches for threshold selection are hugely limited by the available data types. There are many methods to select the threshold for presence‐absence data (Liu et al., 2005). However, since determining whether a species is truly absent or simply not detected is difficult, absence data are hard to obtain and often unreliable (Della Rocca et al., 2020; Gomes et al., 2018; Guillera‐Arroita et al., 2015). It is problematic to select a suitable threshold for presence‐only data, as conventional confusion matrices cannot be formed (Liu et al., 2013; Rebelo & Jones, 2010). A prevalent method to choose thresholds is fixed sensitivity, as it can be conveniently calculated from presence‐only data. Some common fixed values include sensitivities equal to 0.5, 0.75, and 0.9 (Se0.5, Se0.75, and Se0.9) (Bellamy et al., 2013; Ensing et al., 2013; Toth et al., 2013). The selection of these parameters, however, is totally subjective and lacks theoretical justification (Liu et al., 2013). Other researchers use pseudo‐absence data to calculate sensitivity and specificity and then select the threshold. The threshold that equalizes sensitivity and specificity is a common strategy (ESS) (Dobrowski et al., 2011; Swanson et al., 2013), as is the threshold that maximizes the sum (MSS) (Higgins et al., 2012; Vacchiano et al., 2013). Since the determination of specificity is based on pseudo‐absence data rather than real absence data, the deviation between the threshold generated by these methods and the true value cannot be avoided. Another method of threshold selection is to use known traits of species data and predicted values, for example, the mean predicted probability (MeanProb) (Nenzén & Araújo, 2011).

Boyce established the predicted‐expected (P/E) ratio to evaluate the accuracy of species distribution models (Boyce et al., 2002). This method assesses accuracy by calculating the ratio between the predicted frequencies of the model and the expected frequencies of the random distribution without using absence data. Hirzel pointed out that the information in the P/E curve can help to reclassify continuous output results (Hirzel et al., 2006). Some studies set the boundary of P/E = 1 as an objective threshold (Arshad et al., 2012; Witt et al., 2022), but they can only ensure that the transformed prediction results are better than the random results. There are studies suggesting that the threshold can be determined based on the shape of the curve (Folmer et al., 2016; Wang et al., 2008). Compared to setting P/E = 1, this threshold selection method utilizes more curve information but relies on the judgment of researchers to select specific points, which is arbitrary and unrepeatable.

We discovered that mutation points and slope changes are typical shape features of P/E curves after reviewing relevant articles. Then the source‐sink theory was applied to demonstrate that mutation points can serve as effective thresholds for distinguishing high‐quality habitats (in this article, “mutation point” is also widely referred to as the threshold value). Source‐sink pattern is a well‐established concept in landscape ecology and is considered to exist widely in nature (Dunning et al., 1992; Furrer & Pasinelli, 2016; Gilroy & Edwards, 2017; Runge et al., 2006). A “sink” is a specific habitat or patch where species cannot grow naturally and rely on external migration (mortality > natality). And a “source” is a habitat in which species can grow and spread naturally (mortality < natality) (Pulliam, 1988). Many studies have shown that identifying sources and sinks is critical for species conservation research, and production‐capable source points are key areas that need to be protected (Gervasi et al., 2015; Gilroy & Edwards, 2017; Weegman et al., 2016). We believe that as habitat suitability grows, there is a shift in “sink‐source” type at a specific range. This shift is reflected in the P/E curve as an abrupt change in slope at the inflection point.

The method proposed in this paper introduces threshold regression to quantitatively analyze the structural change of the P/E curve and accurately select the mutation point as the threshold. We call this method Boyce‐Threshold Quantile Regression (BTQR). We apply the threshold obtained from BTQR to classify the predictions of different species distribution models, including the generalized additive model (GAM), generalized boosted regression model (GBM), generalized linear model (GLM), random forest (RF), multivariate adaptive regression splines (MARS), and maximum entropy (MAXENT). We designed a comparative experiment using the real species giant panda *Ailuropoda melanoleuca* and four virtual species with prevalences of 0.1, 0.25, 0.5, and 0.75 to evaluate the classification effects of BTQR and other threshold methods. The empirical study results demonstrate that BTQR has a broader applicability, relatively high accuracy, and greater consistency in changes to the validation dataset.

## 2 THEORETICAL EXPLANATION OF BOYCE‐THRESHOLD QUANTILE REGRESSION

### 2.1 The feature of the P/E curve shape

Boyce proposed a method for evaluating species distribution models that requires only presence data (Boyce et al., 2002). The method divides the continuous predictions of the model output into b classes and calculates two metrics for each class: (1) the predicted frequency of validation points $P_{i}$. (2) the expected probability of validation points $E_{i}$, that is, the frequency expected from a random distribution across the study area.

(1) $$P_{i}=\frac{p_{i}}{\sum_{j=1}^{b}p_{j}}$$

(2) $$E_{i}=\frac{a_{i}}{\sum_{j=1}^{b}a_{j}}$$

$p_{i}$ is the number of validation points belonging to class i and $\sum p_{j}$ is the total number of validation points. $a_{i}$ is the number of grid cells or area covered that belongs to class i and $\sum a_{j}$ is the total number of grid cells or total area of the entire study area. Finally, for each class i, the predicted‐to‐expected (P/E) ratio $F_{i}$ is given by

(3) $$F_{i}=\frac{P_{i}}{E_{i}}$$

By using a moving window with a very small width of W, the habitat suitability values covering the range of [0,1] can be divided into 1/W classes. A smooth P/E curve can be plotted against the average suitability value of each class. If the species distribution model correctly predicts species habitat, $F_{i}$ should be positively correlated with the model prediction value, and the P/E curve is expected to show a monotonic increase.

We searched the Web of Science and Google Scholar platforms for articles from 2008 to 2022 using the keywords “P/E curve,” “predicted‐expected ratio,” “Boyce index,” and “species distribution model.” Thirteen articles that plotted P/E curves in studies related to species distribution modeling were screened in the search results. Table 1 summarizes the relevant information from these 13 articles. The review indicates that there may be a universal pattern in the shape of the P/E curve. Except for one study that classified habitat suitability into four categories and could not reflect the explicit shape information of the curve, all other P/E curves are nonlinear. The curves in 11 of the papers exhibit typical concave features with mutation points. The slopes of these curves vary within different intervals, with lower values when habitat suitability is low and significantly higher values when habitat suitability is higher than a certain value.

**TABLE 1:** Summary of literature analyzed in our review, including the name of the species, resolution of the P/E curve, and description of the P/E curve.

Paper	Species	Resolution	Description of P/E curve
Arshad et al. (2012)	Kashmir markhor, *Capra falconeri cashmiriensis*	100 classes	Concave upward with a mutation point
D'Elia et al. (2019)	California condor, *Gymnogyps californianus*	100 classes	Concave upward with a mutation point
Glenn et al. (2017)	Northern spotted owls	Continuous	Concave upward with a mutation point
Jiménez and Soberón (2020)	*Lantana camara* and *Papilio garamas*	100 classes	Concave upward with a mutation point
Kanaji et al. (2015)	Hort‐finned pilot whale, *Globicephala macrorhynchus*	10 classes	Wave shape
Mochizuki et al. (2015)	Crested Ibis, *Nipponia nippon*	10 classes	Concave upward with a mutation point
Moriarty et al. (2021)	Humboldt marten, *Martes caurina humboldtensis*	Continuous	Concave upward with a mutation point
Mugo et al. (2020)	Skipjack tuna, *Katsuwonus pelamis*	20 classes	There are nine curves in total, of which three are almost flat, two are nearly linear, one is concave downward, and three curves with good model indicators are concave upward with a mutation point
Sarkar et al. (2017)	Tiger, *Panthera tigris*	4 classes	No explicit shape information
Shatz et al. (2013)	Asian longhorned beetle, *Anoplophora glabripennis*	100 classes	Concave upward with a mutation point
Van Nieuland et al. (2019)	Eurasian eagle owl, *Bubo bubo*	100 classes	Concave upward with a mutation point
Wang et al. (2008)	Spiny rat, *Niviventer coninga*	100 classes	P/E ratio is log scale. After restoring to linear scale, it is Concave upward with a mutation point
Witt et al. (2022)	Mexican, *spotted owl*	100 classes	Concave upward with mutation points

### 2.2 Ecological explanation of the P/E curve

This paper argues that the wide occurrence of mutation points and slope changes in P/E curves in different studies is not a coincidence. When habitat suitability reaches the threshold, the mutation of curve‐shaped characteristics is likely to correspond to the habitat type of the species. We suggest that this transformation can be explained by the source‐sink theory. The source‐sink theory suggests that the differences in habitat types between source and sink are due to different habitat qualities. And for the correct species distribution model, the predicted value should quantitatively reflect habitat quality (Almasieh & Cheraghi, 2022; Guisan et al., 2017; Rew et al., 2020). There will inevitably be a threshold in the value range of the model outputs. Patches with suitability above this threshold are sources, while those below this threshold are sinks. Although species still seem to present at a very low density in some sink patches, we classify these areas as “non‐existence” or “non‐habitat.” It is due to the fact that, on the one hand, its presence is unstable and not self‐sustaining. This corresponds to a common situation in real environments, where a species is present in a nonhabitat area due to migration or random activities. On the other hand, setting the threshold that classifies all presence points as “presence areas” often results in an excessively high false positive rate, which is not conducive to optimizing the distinction between presence and absence. Source patches with suitability above the threshold are deemed “presence” or “habitat.” The following will demonstrate that this threshold will cause a significant change in the slope of the P/E curve.

**FIGURE 1:**
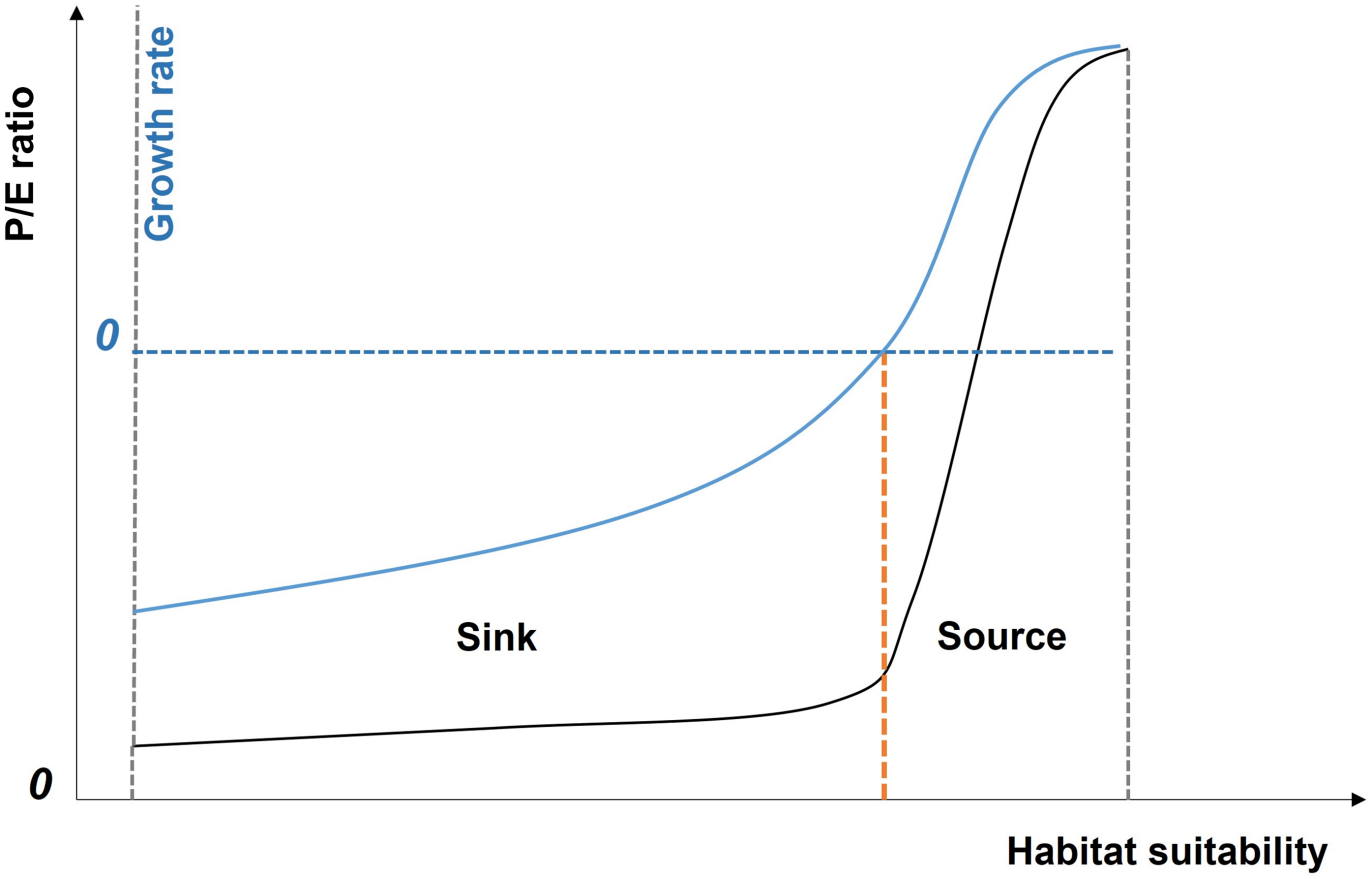
Common pattern of P/E curves summarized from existing literature with a classification framework based on source‐sink theory. The solid black line represents the P/E ratio, the solid blue line represents the growth rate, and the orange dashed line represents the threshold corresponding to the curve mutation point.

The predicted‐expected ratio represents the density of presence points within a certain suitability range, which is positively correlated with the number of individuals in the region. When the suitability is below the threshold, the corresponding habitats are considered sinks. Since all individuals in the sink habitat come from external migration, an increase in suitability within this range will only enhance the attractiveness of the habitat to migrants and cannot increase the natural growth rate. Given that the attractiveness of sink habitats is also restricted by migratory distance and migration resistance, the increase in suitability of sink habitats has a slight effect on the number of species, resulting in a low slope on the P/E curve (Figure 1). When the suitability increases beyond the threshold, the corresponding habitat is the source. The increase in suitability of the source habitat can simultaneously lead to an increase in natural growth rate and attractiveness to migrants. The change in this increasing mechanism greatly increases the correlation coefficient between suitability and presence quantity, causing a significant increase in the slope value of the P/E curve on the right side of the threshold. When suitability reaches a high value, the main constraints limiting the population of the species in the region shift from external environmental factors to intraspecific competition and the animal's reproductive capacity, at which point the P/E ratio no longer increases with suitability and the slope of the curve slows down.

### 2.3 Calculating mutation points through threshold regression

In order to accurately identify mutation points in the structure of the P/E curve, this paper introduces a threshold regression method to avoid the arbitrariness of manual interpretation and improve accuracy. Threshold regression is an extension of traditional linear regression widely used in econometrics (Tong, 1983). This method allows coefficients to vary within different regions, which are identified by threshold variables. A grid search method is used to estimate the value of the threshold, which evaluates the sum of the squared residuals for different values of the threshold parameter and chooses the one that minimizes it.

In this paper, we construct a threshold regression for classifying the output results of species distribution models. This formula is written as

(4) $$\begin{array}{cc} {\mathrm{pe}}_{t}={\mathrm{hs}}_{t}\delta_{1}+\epsilon_{t} & \text{if}\;0<{\mathrm{hs}}_{t}\leq \gamma \\ {\mathrm{pe}}_{t}={\mathrm{hs}}_{t}\delta_{2}+\epsilon_{t} & \text{if}\;\gamma <{\mathrm{hs}}_{t}\leq {\mathrm{hs}}_{\max} \end{array}$$

where pe represents the predicted‐expected ratio as the dependent variable, hs represents habitat suitability predicted by the model as an exogenous variable with region‐specific coefficients $\delta_{1}$ and $\delta_{2}$. $\epsilon_{t}$ is an independent and identically distributed error with mean of 0 and a variance of $\sigma^{2}$. The estimated threshold ($\hat{\gamma }$) is one of the values in the threshold variable ${\mathrm{hs}}_{t}$. To estimate the threshold, we minimize the least squares of the following regression with T observations and two regions,

(5) $${\mathrm{pe}}_{t}={\mathrm{hs}}_{t}\delta_{1}I\left(0<{\mathrm{hs}}_{t}\leq \gamma \right)+{\mathrm{hs}}_{t}\delta_{2}I\left(\;\gamma <{\mathrm{hs}}_{t}\leq {\mathrm{hs}}_{\max}\right)+\epsilon_{t}$$

for a sequence of $T_{1}$ values in ${\mathrm{hs}}_{t}$, where $T_{1}$ < T. The trimming percentage is set to 10%, which implies that $T_{1}$ corresponds to the number of observations between the 10th and the 90th percentile of ${\mathrm{hs}}_{t}$. The formula for estimating the threshold is

(6) $$\hat{\gamma }=\arg\min_{\gamma \epsilon \Gamma }S_{T_{1}}\left(\gamma \right)$$

where $\Gamma =\left(0,\;{\mathrm{hs}}_{\max}\right)$,

(7) $$S_{T_{1}}\left(\gamma \right)=\sum_{t=1}^{T}{\left({\mathrm{pe}}_{t}-{\mathrm{hs}}_{t}\delta_{1}I\left(0<{\mathrm{hs}}_{t}\leq \gamma \right)-{\mathrm{hs}}_{t}\delta_{2}I\left(\;\gamma <{\mathrm{hs}}_{t}\leq {\mathrm{hs}}_{\max}\right)\right)}^{2}$$

is a $T_{1}\times 1$ vector of the sum of the squared differences between the observed predicted‐expected ratio and the estimated value, and γ is a $T_{1}\times 1$ vector of tentative thresholds.

## 3 MATERIALS AND METHODS

We use both virtual species simulations and empirical studies to systematically analyze the validity and applicability conditions of different threshold methods. The virtual species approach can generate simulated distribution data with different prevalences by adjusting the response function to environmental factors, conveniently assessing the sensitivity of threshold methods to different combinations of model and species traits. And the spatially explicit simulated data also make it possible to compare the classified binary output to a known (virtual) “truth” (Miller, 2014). Most of the existing studies used virtual species to evaluate the effectiveness of threshold selection methods (Li & Guo, 2013; Liu et al., 2013, 2016). However, given that artificially set affecters and response functions can hardly depict the complex and ambiguous relationships between species and natural environments, assessment with only virtual species does not fully reflect the effectiveness of the method in the real world (Zurell et al., 2010). We therefore used the giant panda, a real species, as a supplement to the virtual species simulation.

**FIGURE 2:**
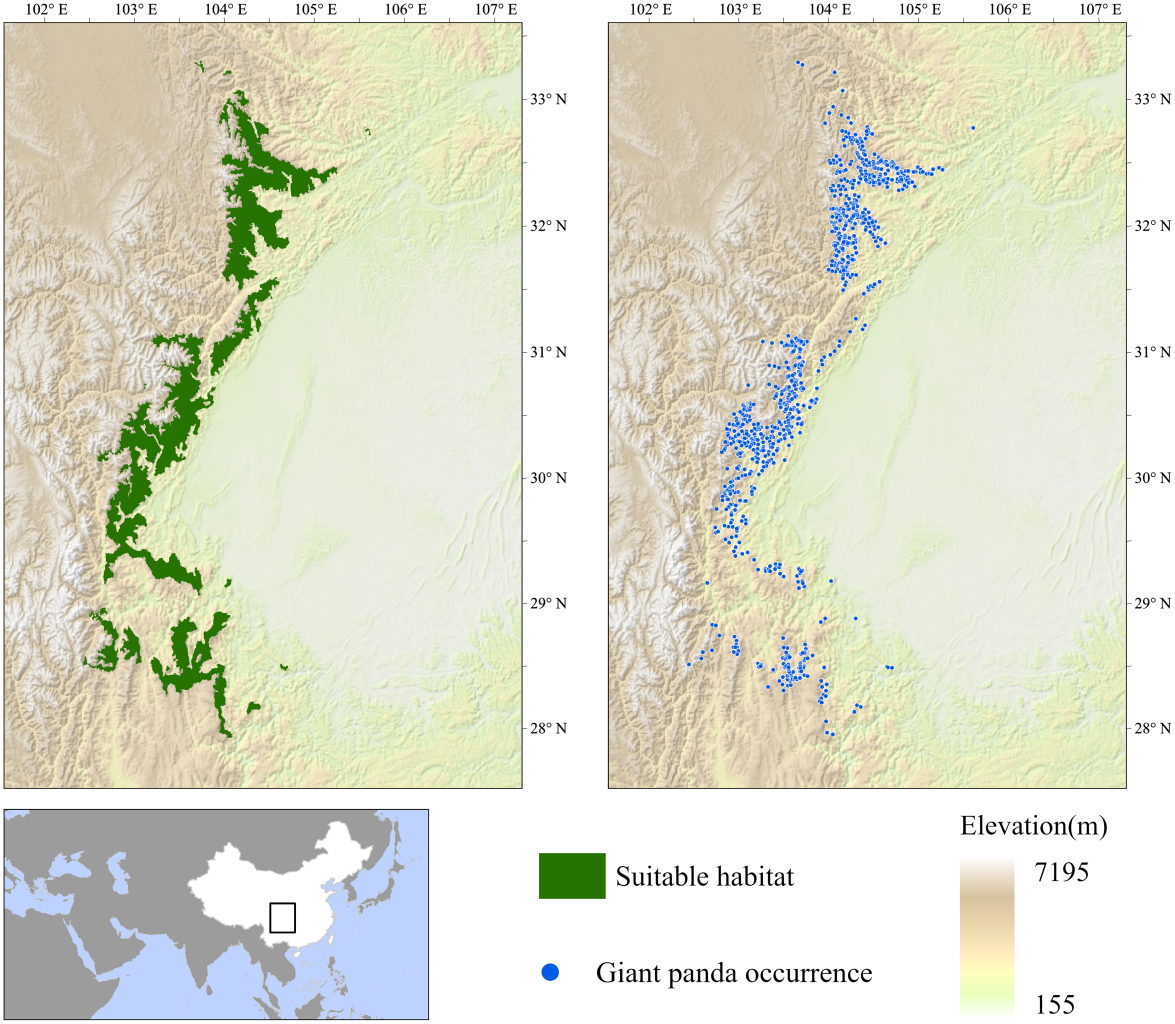
Suitable Habitat (green patches) and Location of Giant Pandas (blue dots) Recorded by the 4th Survey on Giant Panda in Sichuan Province, China.

For real species research, we used data from the Chinese government's 4th Giant Panda Survey on giant panda occurrences and suitable habitat in Sichuan Province, China. The 4th Survey on Giant Panda between 2011 and 2014 covered 42 counties in Sichuan Province, with 13,737 survey lines, including all habitats and potential habitats identified in previous surveys, areas where habitat conditions may be suitable, and areas where pandas may be found based on reports from residents (Jiang et al., 2015). During the sampling process, the survey areas were divided into grids with reference to the activity habits of giant pandas and the size of the nest area. Differences in the travel distance and length of giant panda scat sections were used to distinguish whether the observations were of the same panda. The above design minimizes sampling bias in the survey process (Tang et al., 2015). Researchers in this survey also assessed the status of habitat topography, vegetation, food, and co‐distributed wildlife resources. The assessment results were used to analyze the suitability for giant panda survival and map the distribution of suitable habitat for giant pandas in Sichuan Province (Tang et al., 2015) (Figure 2). This provided suitable evaluation data for this study to construct confusion matrices against threshold classification results for accuracy assessment.

### 3.1 Data description

The real species modeling used 910 occurrences marked by the 4th Survey on Giant Panda in Sichuan Province. We chose the same study area for modeling based on virtual and real species. The study area was selected as the main distribution area for wild giant pandas in Sichuan Province, with a total area of approximately 318,175 km^2^ at latitudes 27.80° to 34.09° N and longitudes 100.80° to 106.60° E (Figure 2).

For both virtual species and real species, three sets of environmental predictor variables were used in this study: climatic data, topographic data, and population density data. The climatic data were obtained from the global climate dataset provided by the WorldClim website (at https://www.worldclim.org/data/) at a resolution of 30″. This dataset consists of 19 climate factors related to biological activity, such as monthly temperature (minimum, maximum, and average), precipitation, solar radiation, barometric pressure, and wind speed (Fick & Hijmans, 2017). Topographic data are composed of digital elevation models (DEM) and slope, with DEM data from the ASTER GDEM V3 dataset published by NASA (Abrams et al., 2020) and slope calculated from elevation data. Population density data were obtained from the 1‐km resolution population density data published on the WorldPop website (at https://www.worldpop.org/datacatalog/) (Tatem, 2017). The boundaries of the environmental variable data layers were unified. The raster size of all environmental factors was set to 1 km × 1 km by resampling. Principal component analysis was performed on a total of 22 environmental factors to reduce data redundancy and improve data processing efficiency, resulting in three principal component layers.

### 3.2 Generation of virtual species

The virtual species were created in three steps (Leroy et al., 2016). First, the environmental suitability was calculated using an additive approach to the response functions of each principal component, where the possible types of response functions implemented are “gaussian,” “linear,” and “logistic.” The obtained environmental suitability was then rescaled between 0 and 1 (i.e., the range of possible probability values of the virtual species distribution). Second, we convert the environmental suitability raster into a probability of occurrence with a logistic transformation setting *α* and *β* parameters that determine the shape of the logistic curve. Finally, each grid cell in the landscape was subjected to a Bernoulli trial by simply generating a random number *x* out of a uniform distribution between 0 and 1, with which to compare its probability Pr. We assigned a pixel presence (*y* = 1) if *x* < Pr and absence (*y* = 0) otherwise, resulting in the realized presence‐absence map.

We generated four types of species with different preferences for environmental factors by setting the type, arrangement, and parameter values of the response function (see Appendix S1). To investigate the sensitivity of the thresholding approach to species prevalence, we set the *α* value of logistic conversion to −.05 and adjusted the *β* value to set the prevalence of the above species to 0.1, 0.25, 0.5, and 0.75, respectively.

### 3.3 Model building

In order to compare the effectiveness of the thresholding method for different species distribution models and to ensure the generalizability of the BTQR method, six species distribution models, including the generalized linear model (GLM), generalized additive model (GAM), generalized boosted regression model (GBM), random forest (RF), multivariate adaptive regression splines (MARS), and maximum entropy (MAXENT), were used in this study (see the tuning parameters in Appendix S2). GLM, GAM, GBM, and MARS are regression‐based models; RF is an integrated learning model based on decision trees; and MAXENT is a machine learning model that estimates species distribution by finding the distribution with maximum entropy (i.e., closest to geographic homogeneity). In this study, GAM, GBM, GLM, RF, and MARS models were constructed through the Biomod2 package of the R language (Thuiller et al., 2009), and MAXENT models were constructed through Maxent software (Phillips et al., 2006).

For virtual species modeling, we sampled presence points in the created presence‐absence map for the training dataset. After excluding the presence points used for modeling, background points were randomly extracted as pseudo‐absence data throughout the study area. Based on the recommendations of previous studies, we fixed the ratio of presence to absence points in the training set to 1:2 for models built with absence data (GAM, GBM, GLM, MARS, and RF) (Barbet‐Massin et al., 2012; Liu et al., 2016). We constructed training sets for the GAM, GBM, GLM, MARS, and MAXENT models. For species prevalence of 0.1 or 0.25, we utilised 5000 presence points, whereas for prevalence of 0.5 or 0.75, we employed 10,000 presence points. For the RF model, since using too many random points can lead to a significant decrease in model accuracy, we sampled 50 presence points at species prevalences of 0.1, 0.25, and 100 presence points at species prevalences of 0.5, 0.75. All of the above sampling and training processes were iterated 10 times by independent procedures to reduce sampling bias.

For real species modeling, to reduce spatial autocorrelation between data, we excluded points that were too close in geographic space and screened 300 out of 910 presence points of giant pandas as training data. The resolution to rarefy data was set to 4 km, considering the average nesting area size of giant pandas (Zhu et al., 2010). MAXENT is trained using presence‐only data. For GAM, GLM, RF, and MARS, we randomly selected 5000 background data points as pseudo‐absence data in the same way as virtual species and combined them with the presence data mentioned above to create a presence‐absence dataset for model training. The model creation process for real species is iterated 10 times through 10‐fold cross validation.

### 3.4 Threshold selection

Ten threshold selection methods were applied in this comparative study, including: (1) maximizing the sum of sensitivity and specificity (MSS), (2) equalizing sensitivity with specificity (ESS), (3) maximizing kappa (MaxKappa), (4) Boyce‐Threshold Quantile Regression (BTQR), (5) equalizing predicted prevalence with observed prevalence (equalPrev), (6) mean predicted probability (MeanProb), (7) minimizing distance between the receiver operating characteristic (ROC) curve plot and the point (0,1) (MinROC), (8) equalizing sensitivity with 0.5 (Se0.5), (9) equalizing sensitivity with 0.75 (Se0.75), and (10) equalizing sensitivity with 0.9 (Se0.9). Validation datasets with different numbers of presence points were generated to evaluate the effect of the presence amount on the threshold classification accuracy. For the virtual species, we generated validation datasets with 1000, 2000, 4000, and 8000 presence points by an independent sampling procedure and subsequently sampled 10,000 background points as pseudo‐absence data in the dataset. For real species, validation datasets containing 100, 200, 300, and 400 presence points were constructed, with 1000 background points acting as pseudo‐absences. Both presence and pseudo‐absence data in each group of validation datasets were randomly sampled 10 times to limit the impact of sample bias on the experiment and to test the stability of different thresholding strategies for random validation datasets. Threshold selection methods (1)~(3) use both presence and pseudo‐absence data, while threshold selection methods (4)~(10) are based on presence data only.

### 3.5 Threshold method assessment

We assessed the accuracy of the threshold methods using kappa values. For virtual species, we created test datasets based on species prevalence. The test dataset for each virtual species was obtained by randomly sampling from previously generated presence‐absence maps, including 50,000 *p* presence points and 50,000 (1−*p*) absence points (*p* is the prevalence of virtual species). We compared the classification results of these points with the true labels, calculating the kappa values. For real species, we calculated Kappa values by comparing the binary maps after threshold classification with the habitat distribution maps assessed by the 4th Survey on Giant Panda.

We assessed the consistency of the threshold method in the changes to the validation dataset through the *F*‐test. For each threshold method, we classify the calculated thresholds into four groups based on the validation dataset used. We consider the validation dataset as the independent variable and compute the *F*‐value, which can be used to test the consistency of the means of multiple sets of data. The higher the *F*‐value, the greater the likelihood that the means of threshold groups are different, which also means that the corresponding threshold method is more likely to be affected by the number of presence points in the validation dataset. Considering that the number of groups and samples in the validation datasets set up in the experiments for each species are identical, the *F*‐values of the various types of threshold methods can be directly compared. The *p*‐value obtained from the hypothesis test was used to assess whether there was a difference in the mean values of the threshold groups.

## 4 RESULTS

**FIGURE 3:**
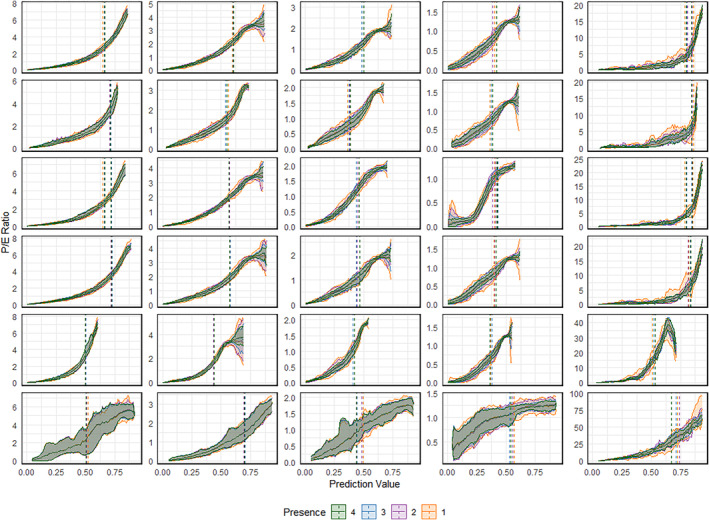
The ratio of predicted‐expected (P/E) frequency versus prediction values of the species distribution model. Each column of subplots from left to right corresponds to virtual species 1–4 and real species Giant Panda. Each subplot row from top to bottom corresponds to the models GAM, GBM, GLM, MARS, MAXENT, and RF. Different colors of “presence” correspond to different numbers of presence points in the validation dataset. For virtual species, Presence 1–4 corresponds to the number of presence points: 1000, 2000, 4000, and 8000. For real species, Presence 1–4 corresponds to the number of presence points: 100, 200, 300, and 400. The light ribbon reflects the range of fluctuations in P/E across all stochastic realizations at the 90 percent confidence level. The dark solid line in the middle of the light band is the mean value of P/E. The dashed line perpendicular to the *x*‐axis represents the mean threshold calculated by the BTQR method.

Figure 3 illustrates the P/E curves and the corresponding thresholds calculated based on the BTQR method for a moving window resolution of 100. All curves show a monotonically increasing trend. For species 1 and the giant panda, the shape of the curves is upward concave exponential. For species 2, 3, and 4, the shape of the curves is mostly closer to a sigmoid curve, with a slope that is small at the beginning, then increases abruptly, and finally flattens out again. From species 1 to 4, the relative position of the thresholds calculated by the BTQR method on the curve's changes with species prevalence but is always located near the point where the slope mutates. The shape of the P/E curve and the position of the thresholds hardly change when, all else being equal, the validation dataset changes. From one randomized experiment to the next, the curve shifts up and down within a certain range, but the shape of the curve corresponding to the same set of models remains essentially unchanged. The number of presence points in the validation dataset has an effect on the magnitude of the fluctuations; the greater the number of presence points, the smaller the range of fluctuations in the curve.

**FIGURE 4:**
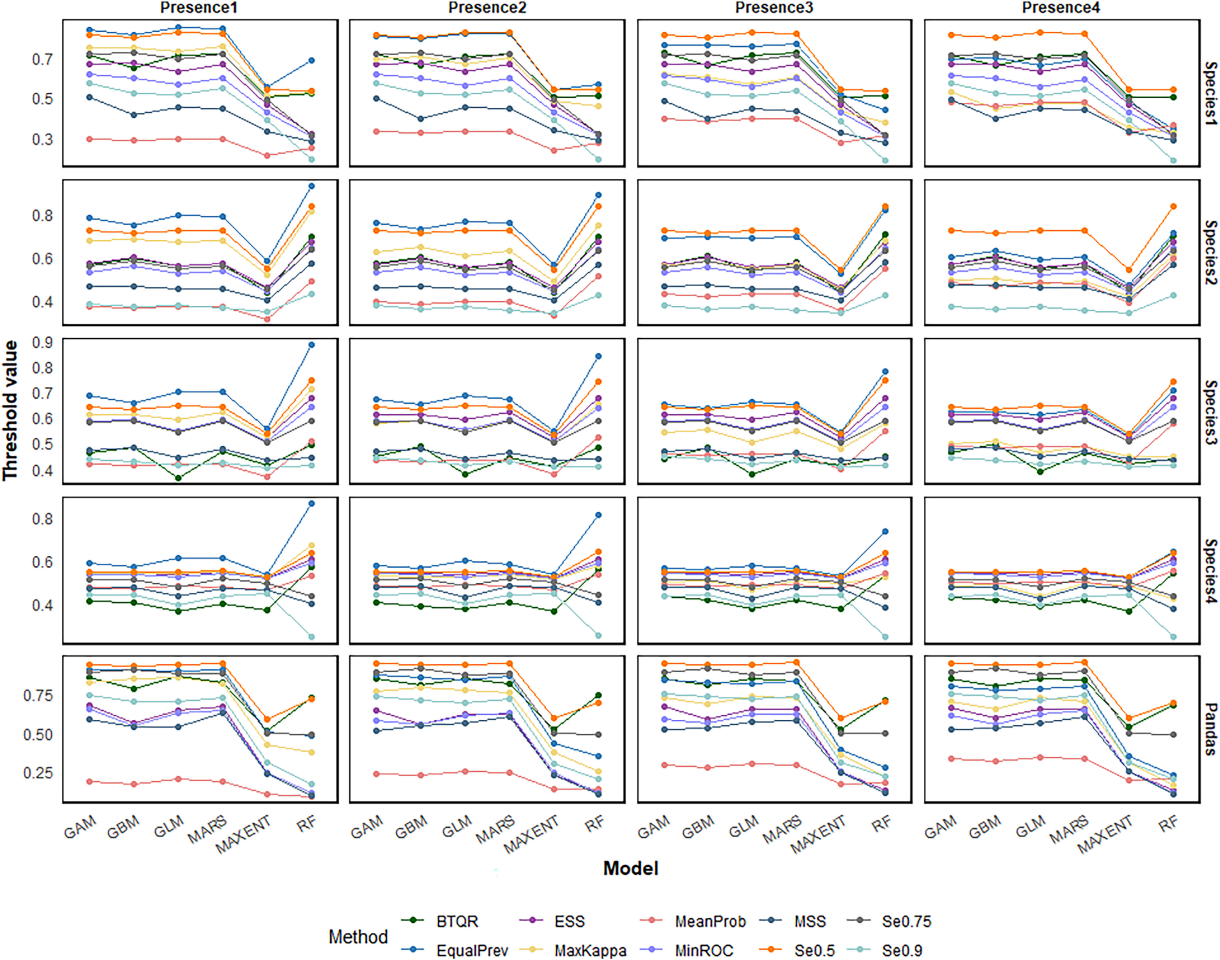
The average threshold values are estimated by different threshold methods. The number at the top of each column represents the quantity of presence points in validation datasets. For virtual species, Presence 1–4 corresponds to the number of presence points: 1000, 2000, 4000, and 8000. For real species, Presence 1–4 corresponds to the number of presence points 100, 200, 300, and 400. Species 1–4 correspond to species prevalences of 0.1, 0.25, 0.5, and 0.75 in order.

Figure 4 illustrates the average threshold values estimated by the different threshold methods. It can be noticed that when the species prevalence and modeling methods change, the value and relative rank of the thresholds calculated by BTQR also change. But when other conditions are fixed, the threshold values estimated by BTQR are almost unaffected by changes in the number of presence points in the validation dataset.

**FIGURE 5:**
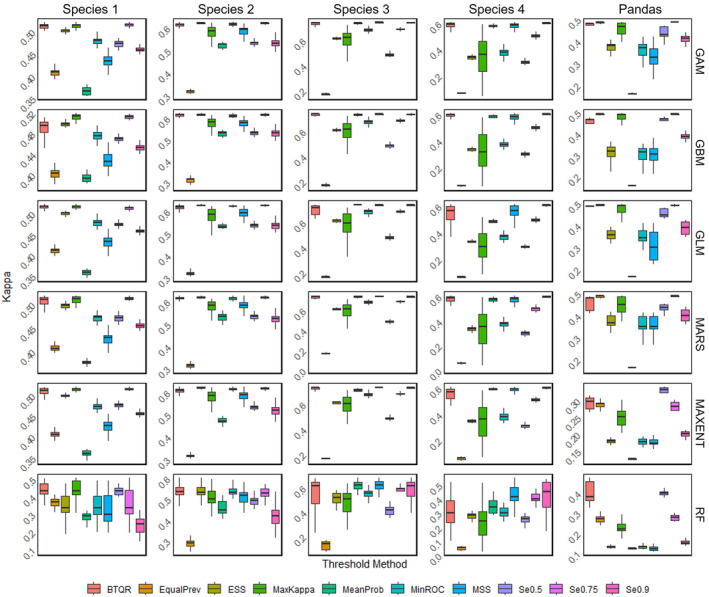
The box plot of kappa values for models classified by different threshold methods with the given validation dataset, “Presence 1.” For virtual species 1–4, the number of presence points is 1000. For the real species giant panda, the number of presence points is 100. The models corresponding to each row are, in turn, GAM, GBM, GLM, MARS, MAXENT, and RF. Box plots of the corresponding kappa values for validation datasets 2–4 are found in Appendix S3.

Figure 5 and Appendix S3 show the distribution of kappa for different threshold methods after classifying the predictions in multiple scenarios with different species, models, and validation datasets in combination with each other. The value of kappa reflects the accuracy of the thresholding methods.

The accuracy of traditional approaches changes significantly when the species prevalence and number of presence points in the validation dataset transition. For EqualPrev, if the quantity of presence points in the validation set is limited, the kappa value is low, but it grows dramatically as the number of presence points increases. The ESS approach performs reasonably well at species prevalences of 0.1 and 0.25, but poorly at higher species prevalences. For MaxKappa, the kappa value decreases with the number of presence points when the species prevalence is 0.1, whereas it increases with the number of presence points at other species prevalences. The kappa value of MeanProb increases with species prevalence. For MinROC, the kappa value is higher at species prevalences of 0.25 and 0.5 and lower at species prevalence of 0.1 and 0.75. The kappa value of MSS is low at a species prevalence of 0.1 and otherwise performs well. For the three fixed sensitivity threshold methods, Se0.5 and Se0.75 are more accurate at low species prevalence, while Se0.9 performs effectively at high species prevalence. For the real species giant panda, the volatility in the accuracy ranking of traditional threshold methods can be affected by the modeling method; for example, EqualPrev possesses a relatively high kappa value on the GAM, GBM, GLM, and MARS models but has low accuracy on the MAXENT and RF models. The performance of all types of threshold methods on giant pandas is generally closer to Species 1, which may be due to the low prevalence of giant pandas as a vulnerable species in reality. However, there are exceptions; for example, the kappa value of ESS is high for Species 1 but low for the giant panda. The kappa value of BTQR, while not exceeding those of other methods in every assessment, stabilizes at a high level in the vast majority of cases (except for the RF model at a species prevalence of 0.75). Unlike traditional methods, whose accuracy declines sharply under some conditions, the accuracy of the BTQR method is virtually unaffected by species prevalence, the modeling approach, or the validation dataset.

**FIGURE 6:**
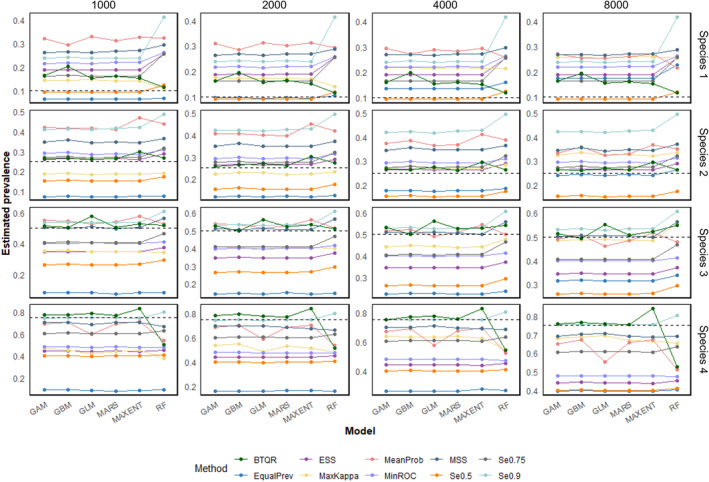
The line plots of species prevalence were estimated based on different models and threshold methods. The number at the top of each column indicates the number of presence points in the validation dataset, from left to right: 1000, 2000, 4000, and 8000. Each row corresponds to a virtual species with a specific prevalence. Species 1–4 correspond to species prevalences of 0.1, 0.25, 0.5, and 0.75 in order. The black dashed lines correspond to the true prevalence of each species. The colors of the line correspond to different threshold methods. The horizontal coordinates of the solid points correspond to different models.

Figure 6 compares the species prevalence estimated based on the various models and threshold methods with the true species prevalence. At a species prevalence of 0.1, the BTQR estimate yields a prevalence slightly higher than the true value, while Se0.5 is close to the true value. Nevertheless, the BTQR estimation is not significantly distant from the true prevalence at this time, and its accuracy is second only to Se0.5. At species prevalences of 0.25 and 0.5, the predicted prevalence based on BQTR is nearly identical to the true prevalence. At a species prevalence of 0.75, the BTQR method performs effectively on all models except RF. Overall, the BTQR method is able to calculate species prevalence with top‐ranked accuracy in most scenarios. In contrast, traditional methods, while some can perform well under specific conditions, are unable to adapt to the situation after changes in species and validation datasets.

**FIGURE 7:**
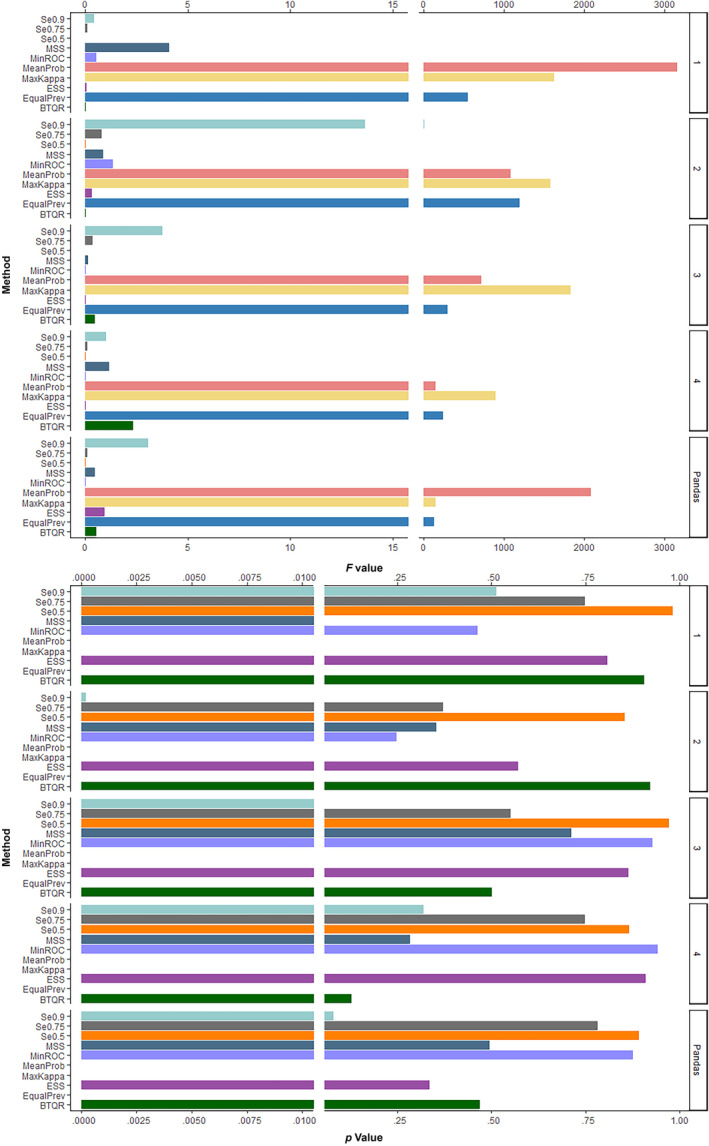
Histograms of the *F*‐value and *p*‐value for each type of threshold method at different species prevalences. The numbers 1, 2, 3, and 4 on the rightmost side of the image represent the species prevalences of 0.1, 0.25, 0.5, and 0.75, respectively.

Figure 7 illustrates the *F* and *p* values corresponding to each type of threshold method when species prevalence changes. MeanProb, MaxKappa, and EqualPrev have *F*‐values in the thousands, considerably higher than the other thresholding methods. Their *p*‐values are also much less than .01. It can be assumed that the thresholds predicted by these three methods are statistically significantly different when the validation datasets are different. The *F*‐values for the BTQR method are much less than one in the real species case, and for species prevalence of 0.1, 0.25, and 0.5, with *p*‐values of .906, .924, .501, and .467, respectively. The thresholds computed by the BTQR have a relatively high degree of consistency among the various sets of thresholds, indicating that the BTQR approach is resistant to the variation of the validation dataset.

## 5 DISCUSSION

### 5.1 Comparison of threshold selection methods

There are two main purposes for transforming continuous predictions of species distribution models into binary outputs through thresholds. The first is to perform the most accurate habitat classification possible. This accuracy can be evaluated by two independent criteria. On the one hand, the classified habitat should contain as many presence sites as possible and exclude as many absence sites as possible. On the other hand, the classified habitat should be close to the habitat range obtained from reliable actual surveys. Previous research employing just virtual species could only evaluate the threshold method based on the first criterion. This study, however, used real giant panda distribution data in addition to virtual species, and thus the effectiveness of the threshold method could be evaluated on two levels simultaneously. The comparison result of the kappa value shows that the commonly used traditional threshold methods only perform well under certain conditions. When relevant factors such as species prevalence, validation datasets, and modeling methods are changed, the accuracy of these methods fluctuates considerably. In contrast, the BTQR method proposed in this paper maintains high accuracy under almost all combinations of conditions. And the kappa value of the BTQR method can be ranked among the top of all methods for both virtual and real species.

The second purpose was to assess species‐related parameters with the help of the binarized result. We generated virtual species with defined species prevalence. By comparing the gap between the threshold estimated prevalence and the true prevalence, we evaluated the effectiveness of each threshold method in predicting this parameter. In most instances, the difference between the BTQR projected prevalence and the real prevalence was rather minimal, except when applying the RF model at 0.75 species prevalence. Other traditional approaches, on the other hand, might be considerably impacted by species prevalence, validation datasets, and modeling methods. The validity of these methods is inconsistent, and their applicability is significantly narrower than that of BTQR.

Liu has summarized three criteria (objectivity, equality, and discriminability) for evaluating threshold methods when only presence data are available (Liu et al., 2013). First, thresholds should be objective rather than arbitrary (e.g., thresholds like MSS and ESS that optimize a statistical indicator). BTQR meets this criterion because it objectively selected the threshold using threshold regression, which determines the mutation point on the curve by minimizing the sum of residual squares. Second, whether we are using presence‐absence data or presence‐only data, the threshold should be identical. As a threshold method that only requires the use of presence data, BTQR satisfies this requirement. Third, the distinction between presence and absence rather than between presence and random point should be optimized. Our experiments have shown that the threshold of BTQR can obtain higher kappa values, meeting the criterion.

In addition to the existing principles, we suggest that a reliable threshold should be stable when using different random validation data. We divided the thresholds obtained by each method into four groups based on the validation dataset used, then performed *F*‐tests. The results show that the thresholds obtained by MeanProb, MaxKappa, and EqualPrev are significantly different when the validation dataset is changed. For thresholds calculated by MSS and Se0.9, the resistance to the variation of the validation dataset depends on species type. For example, thresholds of Se0.9 keep steady at 0.1 species prevalence and vary between each group at 0.2 species prevalence. The threshold consistency obtained for Se0.75, Se0.5, ESS, MinROC, and BTQR was relatively high in all species instances.

### 5.2 Relationship between BTQR and species prevalence, modeling methods

Although the BTQR method generally showed good stability and accuracy across species prevalence, the line shape of the P/E curves and the relative position of the estimated thresholds showed differences. At a species prevalence of 0.1, most of the P/E curves showed an exponential shape, with the BTQR‐estimated thresholds at the inflection points of the curves, and the slopes on the right side of the thresholds were substantially higher compared to the left side. At species prevalence levels of 0.25, 0.5, and 0.75, the P/E curves are more similar to the sigmoid curve. Most of the BTQR threshold positions at high species prevalence will be closer to the end of the curve, and the slope of the right side of the threshold will even be lower than the left side for some models (e.g., GLM at 0.75 prevalence). These differences suggest that the BTQR method, although possessing generality, is not a perfect fit for all species at all prevalence levels. For species with different prevalences, adjusting the parameters and objective function according to their curve characteristics may lead to more accurate thresholds.

The shape of the P/E curve is also affected by the modeling method. Unlike other models, the P/E curves corresponding to the RF model are generally gentler, and the inflection points are not obvious. At a species prevalence of 0.75, the shape of the curve was even concave downward. In this case, the accuracy of the thresholds estimated by BTQR was low, indicating that BTQR could not be applied to this scenario. We suggest that this difference in shape may be due to variations in the decision‐making process of the RF algorithm and the regression model.

## 6 CONCLUSIONS

In conclusion, the Boyce‐Threshold Quantile Regression proposed in this study is a promising method for threshold selection when only presence data are available. The process of selecting thresholds for BTQR is entirely based on objective statistical analysis of presence data, without personal judgment or artificial pseudo‐absence involved. Experiments based on virtual and real species show that the BTQR method can be applied to most combinations of species prevalence and modeling methods. And it is almost unaffected by the number of presence points in the validation dataset. Compared to the current approaches, BTQR is superior in terms of accuracy, applicability, and consistency.

In the present study, we assessed the effectiveness of the BTQR method using five species and six modeling approaches. However, this assessment remains incomplete, and some novel modeling methods, such as INLA‐SPDE (Della Rocca & Milanesi, 2022a, 2022b), were not included in the experiments. In addition, the P/E curves at different species prevalences indicate that there is room for further improvement and refinement of the BTQR method. For species with different prevalences, adjusting the BTQR parameters and objective function according to their curve characteristics may yield more accurate thresholds.

## AUTHOR CONTRIBUTIONS

**Boyang Yu:** Conceptualization (lead); data curation (lead); formal analysis (equal); methodology (lead); writing – original draft (lead); writing – review and editing (equal). **Wenyu Dai:** Data curation (equal); formal analysis (equal); writing – review and editing (equal). **Zhaoning Wu:** Conceptualization (equal); visualization (equal). **Siqing Li:** Data curation (equal); visualization (equal). **Jiechen Wang:** Conceptualization (equal); writing – review and editing (lead).

## CONFLICT OF INTEREST STATEMENT

The authors declare that no conflict of interest exists.

## Supporting information


Appendices S1–S3.

## Data Availability

The simulation data and code are contained in the repository: https://github.com/Izbern/Boyce‐Threshold‐Quantile‐Regression. The presence records and suitable habitat map of giant panda are contained in the repository: https://doi.org/10.5061/dryad.jq2bvq8fb.

## References

[ece311208-bib-0001] Abrams, M. , Crippen, R. , & Fujisada, H. (2020). ASTER global digital elevation model (GDEM) and ASTER global water body dataset (ASTWBD). Remote Sensing, 12, 1156.

[ece311208-bib-0002] Almasieh, K. , & Cheraghi, M. (2022). Habitat suitability, core habitats and diversity hotspots for the conservation of the mustelid species in Iran. Global Ecology and Conservation, 36, e02120.

[ece311208-bib-0003] Araújo, M. B. , Williams, P. H. , & Fuller, R. J. (2002). Dynamics of extinction and the selection of nature reserves. Proceedings of the Royal Society of London. Series B: Biological Sciences, 269, 1971–1980. 10.1098/rspb.2002.2121 PMC1691129 12396495

[ece311208-bib-0004] Arshad, M. , Qamer, F. M. , Saleem, R. , & Malik, R. (2012). Prediction of Kashmir markhor habitat suitability in Chitral Gol National Park, Pakistan. Biodiversity, Tropical Conservancy, 13, 1–10.

[ece311208-bib-0005] Barbet‐Massin, M. , Jiguet, F. , Albert, C. H. , & Thuiller, W. (2012). Selecting pseudo‐absences for species distribution models: How, where and how many? Methods in Ecology and Evolution, 3, 327–338.

[ece311208-bib-0006] Bayliss, S. L. J. , Mueller, L. O. , Ware, I. M. , Schweitzer, J. A. , & Bailey, J. K. (2022). Stacked distribution models predict climate‐driven loss of variation in leaf phenology at continental scales. Communications Biology, 5, 1213. 36357488 10.1038/s42003-022-04131-z PMC9649771

[ece311208-bib-0007] Bellamy, C. , Scott, C. , & Altringham, J. (2013). Multiscale, presence‐only habitat suitability models: Fine‐resolution maps for eight bat species. Journal of Applied Ecology, 50, 892–901.

[ece311208-bib-0008] Boyce, M. S. , Vernier, P. R. , Nielsen, S. E. , & Schmiegelow, F. K. A. (2002). Evaluating resource selection functions. Ecological Modelling, 157, 281–300.

[ece311208-bib-0009] Charney, N. D. , Record, S. , Gerstner, B. E. , Merow, C. , Zarnetske, P. L. , & Enquist, B. J. (2021). A test of species distribution model transferability across environmental and geographic space for 108 Western North American tree species. Frontiers in Ecology and Evolution, 9, 689295.

[ece311208-bib-0010] D'Elia, J. , Brandt, J. , Burnett, L. J. , Haig, S. M. , Hollenbeck, J. , Kirkland, S. , Marcot, B. G. , Punzalan, A. , West, C. J. , Williams‐Claussen, T. , Wolstenholme, R. , & Young, R. (2019). Applying circuit theory and landscape linkage maps to reintroduction planning for California condors. PLoS One, 14, e0226491. 31891594 10.1371/journal.pone.0226491 PMC6938332

[ece311208-bib-0011] Della Rocca, F. , Bogliani, G. , Breiner, F. T. , & Milanesi, P. (2019). Identifying hotspots for rare species under climate change scenarios: Improving saproxylic beetle conservation in Italy. Biodiversity and Conservation, 28, 433–449.

[ece311208-bib-0012] Della Rocca, F. , & Milanesi, P. (2020). Combining climate, land use change and dispersal to predict the distribution of endangered species with limited vagility. Journal of Biogeography, 47, 1427–1438.

[ece311208-bib-0013] Della Rocca, F. , & Milanesi, P. (2022a). The new dominator of the world: Modeling the global distribution of the Japanese beetle under land use and climate change scenarios. Land, 11, 567.

[ece311208-bib-0014] Della Rocca, F. , & Milanesi, P. (2022b). The spread of the Japanese beetle in a European human‐dominated landscape: High Anthropization favors colonization of *Popillia japonica* . Diversity, 14, 658.

[ece311208-bib-0015] Della Rocca, F. , Milanesi, P. , Magna, F. , Mola, L. , Bezzicheri, T. , Deiaco, C. , & Bracco, F. (2020). Comparison of two sampling methods to estimate the abundance of *Lucanus cervus* with application of n‐mixture models. Forests, 11, 1085.

[ece311208-bib-0016] Dobrowski, S. Z. , Thorne, J. H. , Greenberg, J. A. , Safford, H. D. , Mynsberge, A. R. , Crimmins, S. M. , & Swanson, A. K. (2011). Modeling plant ranges over 75 years of climate change in California, USA: Temporal transferability and species traits. Ecological Monographs, 81, 241–257.

[ece311208-bib-0017] Dunning, J. B. , Danielson, B. J. , & Pulliam, H. R. (1992). Ecological processes that affect populations in complex landscapes. Oikos, 65, 169–175.

[ece311208-bib-0018] Elith, J. , & Leathwick, J. R. (2009). Species distribution models: Ecological explanation and prediction across space and time. Annual Review of Ecology, Evolution, and Systematics, 40, 677–697.

[ece311208-bib-0019] Ensing, D. J. , Moffat, C. E. , & Pither, J. (2013). Taxonomic identification errors generate misleading ecological niche model predictions of an invasive hawkweed. Botany, 91, 137–147.

[ece311208-bib-0020] Fick, S. E. , & Hijmans, R. J. (2017). WorldClim 2: New 1‐km spatial resolution climate surfaces for global land areas. International Journal of Climatology, 37, 4302–4315.

[ece311208-bib-0021] Folmer, E. O. , van Beusekom, J. E. E. , Dolch, T. , Gräwe, U. , van Katwijk, M. M. , Kolbe, K. , & Philippart, C. J. M. (2016). Consensus forecasting of intertidal seagrass habitat in the Wadden Sea. Journal of Applied Ecology, 53, 1800–1813.

[ece311208-bib-0022] Franklin, J. (2013). Species distribution models in conservation biogeography: Developments and challenges. Diversity and Distributions, 19, 1217–1223.

[ece311208-bib-0023] Furrer, R. D. , & Pasinelli, G. (2016). Empirical evidence for source–sink populations: A review on occurrence, assessments and implications. Biological Reviews, 91, 782–795. 26010659 10.1111/brv.12195

[ece311208-bib-0024] Gervasi, V. , BRøseth, H. , Nilsen, E. B. , Ellegren, H. , Flagstad, Ø. , & Linnell, J. D. C. (2015). Compensatory immigration counteracts contrasting conservation strategies of wolverines (Gulo gulo) within Scandinavia. Biological Conservation, 191, 632–639.

[ece311208-bib-0025] Gilroy, J. J. , & Edwards, D. P. (2017). Source‐sink dynamics: A neglected problem for landscape‐scale biodiversity conservation in the tropics. Current Landscape Ecology Reports, 2, 51–60.

[ece311208-bib-0026] Glenn, E. , Lesmeister, D. , Davis, R. , Hollen, B. , & Poopatanapong, A. (2017). Estimating density of a territorial species in a dynamic landscape. Landscape Ecology, 32, 563–579.

[ece311208-bib-0027] Gomes, V. H. F. , Ijff, S. D. , Raes, N. , Amaral, I. L. , Salomão, R. P. , de Souza Coelho, L. , de Almeida Matos, F. D. , Castilho, C. V. , de Andrade Lima Filho, D. , López, D. C. , Guevara, J. E. , Magnusson, W. E. , Phillips, O. L. , Wittmann, F. , de Jesus Veiga Carim, M. , Martins, M. P. , Irume, M. V. , Sabatier, D. , Molino, J.‐F. , … Ter Steege, H. (2018). Species distribution modelling: Contrasting presence‐only models with plot abundance data. Scientific Reports, 8, 1003. 29343741 10.1038/s41598-017-18927-1 PMC5772443

[ece311208-bib-0028] Guillera‐Arroita, G. , Lahoz‐Monfort, J. J. , Elith, J. , Gordon, A. , Kujala, H. , Lentini, P. E. , McCarthy, M. A. , Tingley, R. , & Wintle, B. A. (2015). Is my species distribution model fit for purpose? Matching data and models to applications. Global Ecology and Biogeography, 24, 276–292.

[ece311208-bib-0029] Guisan, A. , & Thuiller, W. (2005). Predicting species distribution: Offering more than simple habitat models. Ecology Letters, 8, 993–1009. 34517687 10.1111/j.1461-0248.2005.00792.x

[ece311208-bib-0030] Guisan, A. , Zimmermann, N. E. , & Thuiller, W. (Eds.). (2017). Habitat suitability and distribution models: With applications in R. Cambridge University Press.

[ece311208-bib-0031] Higgins, S. I. , O'Hara, R. B. , Bykova, O. , Cramer, M. D. , Chuine, I. , Gerstner, E. M. , Hickler, T. , Morin, X. , Kearney, M. R. , Midgley, G. F. , & Scheiter, S. (2012). A physiological analogy of the niche for projecting the potential distribution of plants. Journal of Biogeography, 39, 2132–2145.

[ece311208-bib-0032] Hirzel, A. H. , Le Lay, G. , Helfer, V. , Randin, C. , & Guisan, A. (2006). Evaluating the ability of habitat suitability models to predict species presences. Ecological Modelling, 199, 142–152.

[ece311208-bib-0033] Howard, C. , Marjakangas, E. L. , Morán‐Ordóñez, A. , Milanesi, P. , Abuladze, A. , Aghababyan, K. , Ajder, V. , Arkumarev, V. , Balmer, D. E. , Bauer, H. G. , Beale, C. M. , Bino, T. , Boyla, K. A. , Burfield, I. J. , Burke, B. , Caffrey, B. , Chodkiewicz, T. , del Moral, J. C. , Mazal, V. D. , … Willis, S. G. (2023). Local colonisations and extinctions of European birds are poorly explained by changes in climate suitability. Nature Communications, 14, 4304. 10.1038/s41467-023-39093-1 PMC10359363 37474503

[ece311208-bib-0034] Jiang, C. , Wang, H. , & Gu, X. (2015). Giant pandas in Sichuan: Report of the fourth Giant panda survey in Sichuan. Sichuan Science and Technology Press.

[ece311208-bib-0035] Jiménez, L. , & Soberón, J. (2020). Leaving the area under the receiving operating characteristic curve behind: An evaluation method for species distribution modelling applications based on presence‐only data. Methods in Ecology and Evolution, 11, 1571–1586.

[ece311208-bib-0036] Kanaji, Y. , Okazaki, M. , Kishiro, T. , & Miyashita, T. (2015). Estimation of habitat suitability for the southern form of the short‐finned pilot whale (*Globicephala macrorhynchus*) in the North Pacific. Fisheries Oceanography, 24, 14–25.

[ece311208-bib-0037] Leroy, B. , Meynard, C. N. , Bellard, C. , & Courchamp, F. (2016). Virtualspecies, an R package to generate virtual species distributions. Ecography, 39, 599–607.

[ece311208-bib-0038] Li, W. , & Guo, Q. (2013). How to assess the prediction accuracy of species presence–absence models without absence data? Ecography, 36, 788–799.

[ece311208-bib-0039] Liu, C. , Berry, P. M. , Dawson, T. P. , & Pearson, R. G. (2005). Selecting thresholds of occurrence in the prediction of species distributions. Ecography, 28, 385–393.

[ece311208-bib-0040] Liu, C. , Newell, G. , & White, M. (2016). On the selection of thresholds for predicting species occurrence with presence‐only data. Ecology and Evolution, 6, 337–348. 26811797 10.1002/ece3.1878 PMC4716501

[ece311208-bib-0041] Liu, C. , White, M. , & Newell, G. (2013). Selecting thresholds for the prediction of species occurrence with presence‐only data. Journal of Biogeography, 40, 778–789.

[ece311208-bib-0042] Miller, J. A. (2014). Virtual species distribution models. Progress in Physical Geography, 38, 117–128.

[ece311208-bib-0043] Mochizuki, S. , Liu, D. , Sekijima, T. , Lu, J. , Wang, C. , Ozaki, K. , Nagata, H. , Murakami, T. , Ueno, Y. , & Yamagishi, S. (2015). Detecting the nesting suitability of the re‐introduced crested ibis Nipponia nippon for nature restoration program in Japan. Journal for Nature Conservation, 28, 45–55.

[ece311208-bib-0044] Mori, E. , Sforzi, A. , Bogliani, G. , & Milanesi, P. (2018). Range expansion and redefinition of a crop‐raiding rodent associated with global warming and temperature increase. Climatic Change, 150, 319–331.

[ece311208-bib-0045] Moriarty, K. M. , Thompson, J. , Delheimer, M. , Barry, B. R. , Linnell, M. , Levi, T. , Hamm, K. , Early, D. , Gamblin, H. , Gunther, M. S. , Ellison, J. , Prevey, J. S. , Hartman, J. , & Davis, R. (2021). Predicted distribution of a rare and understudied forest carnivore: Humboldt marten (*Martes caurina humboldtensis*). PeerJ, 9, e11670. 34434640 10.7717/peerj.11670 PMC8354145

[ece311208-bib-0046] Mugo, R. , Saitoh, S.‐I. , Igarashi, H. , Toyoda, T. , Masuda, S. , Awaji, T. , & Ishikawa, Y. (2020). Identification of skipjack tuna (*Katsuwonus pelamis*) pelagic hotspots applying a satellite remote sensing‐driven analysis of ecological niche factors: A short‐term run. PLoS One, 15, e0237742. 32817669 10.1371/journal.pone.0237742 PMC7440647

[ece311208-bib-0047] Nenzén, H. K. , & Araújo, M. B. (2011). Choice of threshold alters projections of species range shifts under climate change. Ecological Modelling, 222, 3346–3354.

[ece311208-bib-0048] Newbold, T. (2010). Applications and limitations of museum data for conservation and ecology, with particular attention to species distribution models. Progress in Physical Geography, 34, 3–22.

[ece311208-bib-0049] Phillips, S. J. , Anderson, R. P. , & Schapire, R. E. (2006). Maximum entropy modeling of species geographic distributions. Ecological Modelling, 190, 231–259.

[ece311208-bib-0050] Pinto‐Ledezma, J. N. , & Cavender‐Bares, J. (2021). Predicting species distributions and community composition using satellite remote sensing predictors. Scientific Reports, 11, 16448. 34385574 10.1038/s41598-021-96047-7 PMC8361206

[ece311208-bib-0051] Pulliam, H. R. (1988). Sources, sinks, and population regulation. The American Naturalist, 132, 652–661.

[ece311208-bib-0052] Rebelo, H. , & Jones, G. (2010). Ground validation of presence‐only modelling with rare species: A case study on barbastelles *Barbastella barbastellus* (Chiroptera: Vespertilionidae). Journal of Applied Ecology, 47, 410–420.

[ece311208-bib-0053] Rew, J. , Cho, Y. , Moon, J. , & Hwang, E. (2020). Habitat suitability estimation using a two‐stage ensemble approach. Remote Sensing, 12, 1475.

[ece311208-bib-0054] Runge, J. P. , Runge, M. C. , & Nichols, J. D. (2006). The role of local populations within a landscape context: Defining and classifying sources and sinks. The American Naturalist, 167, 925–938. 10.1086/503531 16615034

[ece311208-bib-0055] Sarkar, M. S. , Krishnamurthy, R. , Johnson, J. A. , Sen, S. , & Saha, G. K. (2017). Assessment of fine‐scale resource selection and spatially explicit habitat suitability modelling for a re‐introduced tiger (*Panthera tigris*) population in central India. PeerJ, 5, e3920. 29114438 10.7717/peerj.3920 PMC5672835

[ece311208-bib-0056] Shatz, A. J. , Rogan, J. , Sangermano, F. , Ogneva‐Himmelberger, Y. , & Chen, H. (2013). Characterizing the potential distribution of the invasive Asian longhorned beetle (*Anoplophora glabripennis*) in Worcester County, Massachusetts. Applied Geography, 45, 259–268.

[ece311208-bib-0057] Swanson, A. K. , Dobrowski, S. Z. , Finley, A. O. , Thorne, J. H. , & Schwartz, M. K. (2013). Spatial regression methods capture prediction uncertainty in species distribution model projections through time. Global Ecology and Biogeography, 22, 242–251.

[ece311208-bib-0058] Tang, X. , Jia, J. , Wang, Z. , Zhang, D. , Yu, B. , Yu, J. , Gong, M. , & Liu, Y. (2015). Scheme design and Main result analysis of the Fouth National Survey on Giant pandas. Forest Resources Management, 1, 11–16.

[ece311208-bib-0059] Tatem, A. J. (2017). Comment: WorldPop, open data for spatial demography. Scientific Data, 4, 830. 10.1038/sdata.2017.4 PMC5283060 28140397

[ece311208-bib-0060] Thuiller, W. , Lafourcade, B. , Engler, R. , & Araújo, M. B. (2009). BIOMOD – A platform for ensemble forecasting of species distributions. Ecography, 32, 369–373.

[ece311208-bib-0061] Tong, H. (1983). Threshold models in non‐linear time series analysis . Lecture notes in statistics, No. 2 1.

[ece311208-bib-0062] Toth, J. P. , Varga, K. , Vegvari, Z. , & Varga, Z. (2013). Distribution of the eastern knapweed fritillary (*Melitaea ornata* Christoph, 1893) (lepidoptera: Nymphalidae): Past, present and future. Journal of Insect Conservation, 17, 245–255.

[ece311208-bib-0063] Vacchiano, G. , Barni, E. , Lonati, M. , Masante, D. , Curtaz, A. , Tutino, S. , & Siniscalco, C. (2013). Monitoring and modeling the invasion of the fast spreading alien Senecio inaequidens DC. In an alpine region. Plant Biosystems, 147, 1139–1147.

[ece311208-bib-0064] Van Nieuland, S. , Baetens, J. M. , Janssen, R. , & De Baets, B. (2019). A validated expert‐based habitat suitability assessment for eagle owls in Limburg, The Netherlands. European Journal of Wildlife Research, 65, 48.

[ece311208-bib-0065] Vaughan, I. P. , & Ormerod, S. J. (2005). The continuing challenges of testing species distribution models. Journal of Applied Ecology, 42, 720–730.

[ece311208-bib-0066] Wang, Y.‐H. , Yang, K.‐C. , Bridgman, C. L. , & Lin, L.‐K. (2008). Habitat suitability modelling to correlate gene flow with landscape connectivity. Landscape Ecology, 23, 989–1000.

[ece311208-bib-0067] Weegman, M. D. , Bearhop, S. , Fox, A. D. , Hilton, G. M. , Walsh, A. J. , McDonald, J. L. , & Hodgson, D. J. (2016). Integrated population modelling reveals a perceived source to be a cryptic sink. Journal of Animal Ecology, 85, 467–475. 26717445 10.1111/1365-2656.12481 PMC4785613

[ece311208-bib-0068] Witt, C. , Davis, R. , Yang, Z. , Ganey, J. , Gutiérrez, R. , Healey, S. , Hedwall, S. , Hoagland, S. , Maes, R. , Malcolm, K. , Sanderlin, J. , Seamans, M. , & Jones, G. (2022). Linking robust spatiotemporal datasets to assess and monitor habitat attributes of a threatened species. PLoS One, 17, e0265175. 35298506 10.1371/journal.pone.0265175 PMC8929618

[ece311208-bib-0069] Zhu, L. F. , Zhan, X. J. , Meng, T. , Zhang, S. N. , & Wei, F. W. (2010). Landscape features influence gene flow as measured by cost‐distance and genetic analyses: A case study for giant pandas in the Daxiangling and Xiaoxiangling Mountains. BMC Genetics, 11, 72. 20653932 10.1186/1471-2156-11-72 PMC2918525

[ece311208-bib-0070] Zurell, D. , Berger, U. , Cabral, J. S. , Jeltsch, F. , Meynard, C. N. , Münkemüller, T. , Nehrbass, N. , Pagel, J. , Reineking, B. , Schröder, B. , & Grimm, V. (2010). The virtual ecologist approach: Simulating data and observers. Oikos, 119, 622–635.

