# Supplementary material for: A new threshold selection method for species distribution models with presence‐only data: Extracting the mutation point of the P/E curve by threshold regression
Source: Ecol Evol. 2024 Apr 1;14(4):e11208. doi: 10.1002/ece3.11208 (PMC10985382; doi:10.1002/ece3.11208)

**Appendix S1** Virtual species related parameters and plots.

Table1-1 The type of function used to generate virtual species.

| Species | Function 1 | Function 2 | Function 3 |
| --- | --- | --- | --- |
| Species 1 | linearFun | dnorm | logisticFun |
| Species 2 | dnorm | logisticFun | linearFun |
| Species 3 | dnorm | linearFun | logisticFun |
| Species 4 | logisticFun | linearFun | dnorm |

Table1-2 The parameters used to generate virtual species.

| Species | Parameters | | | | | | | |
| --- | --- | --- | --- | --- | --- | --- | --- | --- |
|  | Function 1 | | Function2 | | | Function 3 | | |
| Species 1 | -0.59057 | 823.6902 | | 1386.018 | 728.4171 | | -34.2335 | 827.621 |
| Species 2 | 3027.491 | 2385.47 | | -62.1183 | 548.972 | | 0.073574 | 0.05367 |
| Species 3 | 946.85 | 2644.79 | | 1331.08 | 964.78 | | 834.72 | 690.77 |
| Species 4 | 318.045 | 4166.829 | | -0.08593 | 0.9346 | | 926.84 | 593.45 |

| 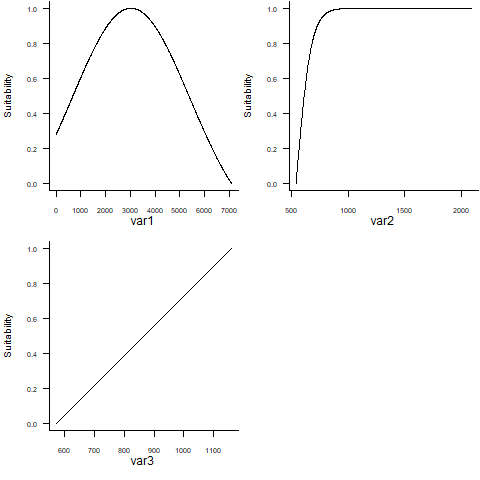  Species 1 | 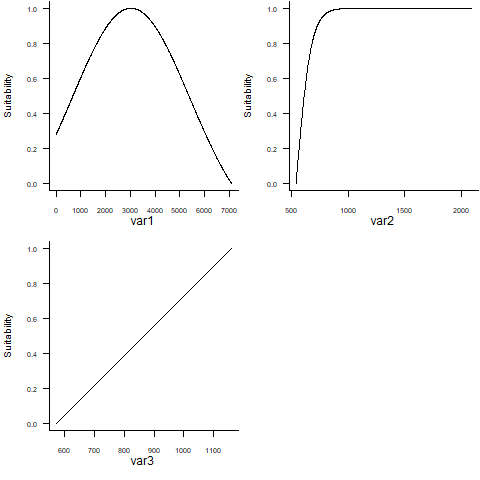  Species 2 |
| --- | --- |
| 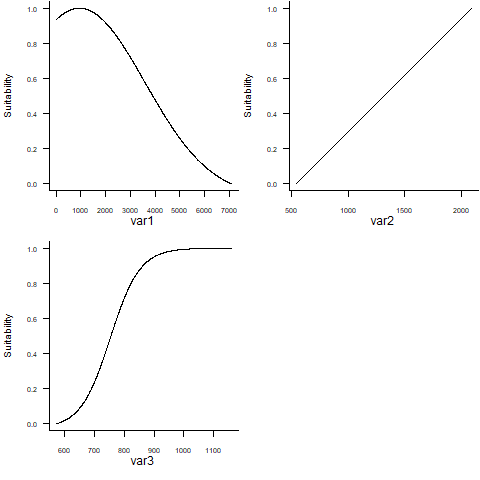  Species 3 | 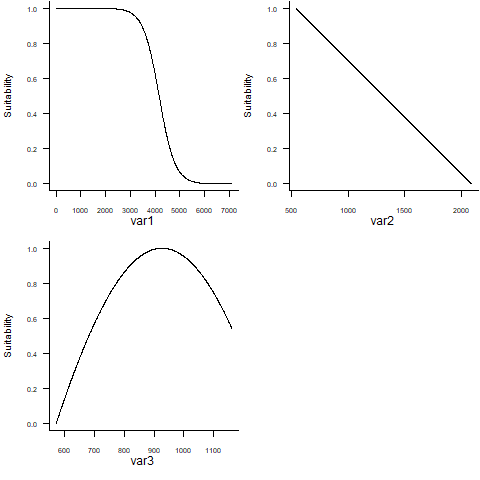  Species 4 |

Figure 1-2 The response curves of virtual species.

| 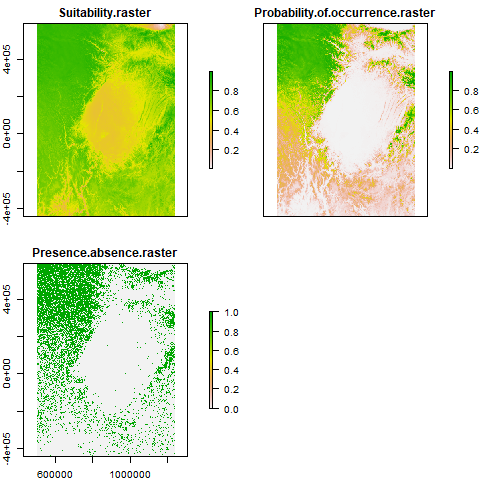  Species 1 | 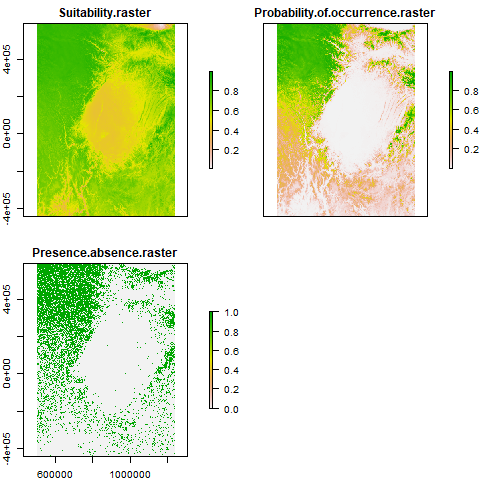  Species 2 |
| --- | --- |
| 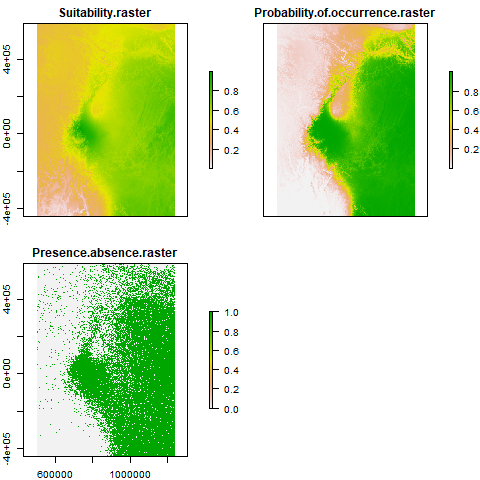  Species 3 | 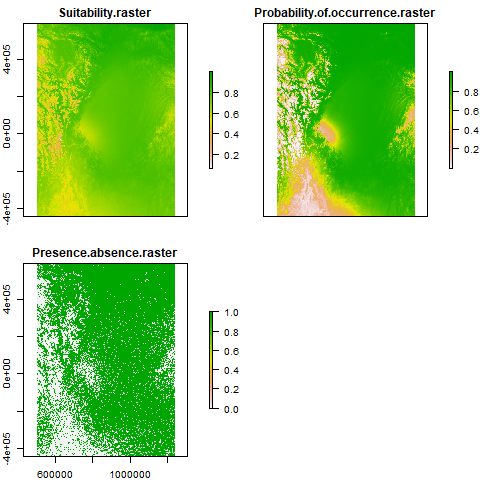  Species 4 |

Figure 2-2 The Suitability raster, probability of occurrence raster and presence-absence raster of virtual species.

**Appendix S2** The tuning parameters for six species distribution models (GAM, GBM, GLM, MARS, MAXENT and RF) used in this study.

| Model | Tuning parameters | | | |
| --- | --- | --- | --- | --- |
| GAM | **algo** | **interaction level** | **smooth term** | **family** |
|  | GAM_mgcv | 0 | -1 | binomial(link = 'logit') |
| GBM | **distribution** | **n.trees** | **interaction.depth** | **n.minobsinnode** |
|  | adaboost | 2500 | 7 | 10 |
|  | **shrinkage** | **cv.folds** | **n.cores** | |
|  | 0.001 | 10 | 8 | |
| GLM | **formula type** | **interaction level** | **test** | **family** |
|  | quadratic | 0 | AIC | binomial(link = 'logit') |
| MARS | **formula type** | **interaction level** | **penalty** | **thresh** |
|  | simple | 0 | 2 | 0.001 |
| MAXENT | **Maximum iterations** | **Regularization multiplier** | **Max number of background points** | |
|  | 500 | 1 | 10000 | |
| RF | **do.classif** | **ntrees** | **nodesize** | **mtry** |
|  | TRUE | 1000 | 1 | 2 |

**Appendix S3** The box plot of kappa values for models classified by different threshold methods with different validation datasets. The number at the top of each column represents the quantity of presence points in validation datasets. For virtual species 1-4, the number of presence points is 1000, 2000, 4000, and 8000 from left to right. For the real species giant panda, the number of presence points is 100, 200, 300, and 400 from left to right. The models corresponding to each row are, in turn, GAM, GBM, GLM, MARS, MAXENT, and RF.


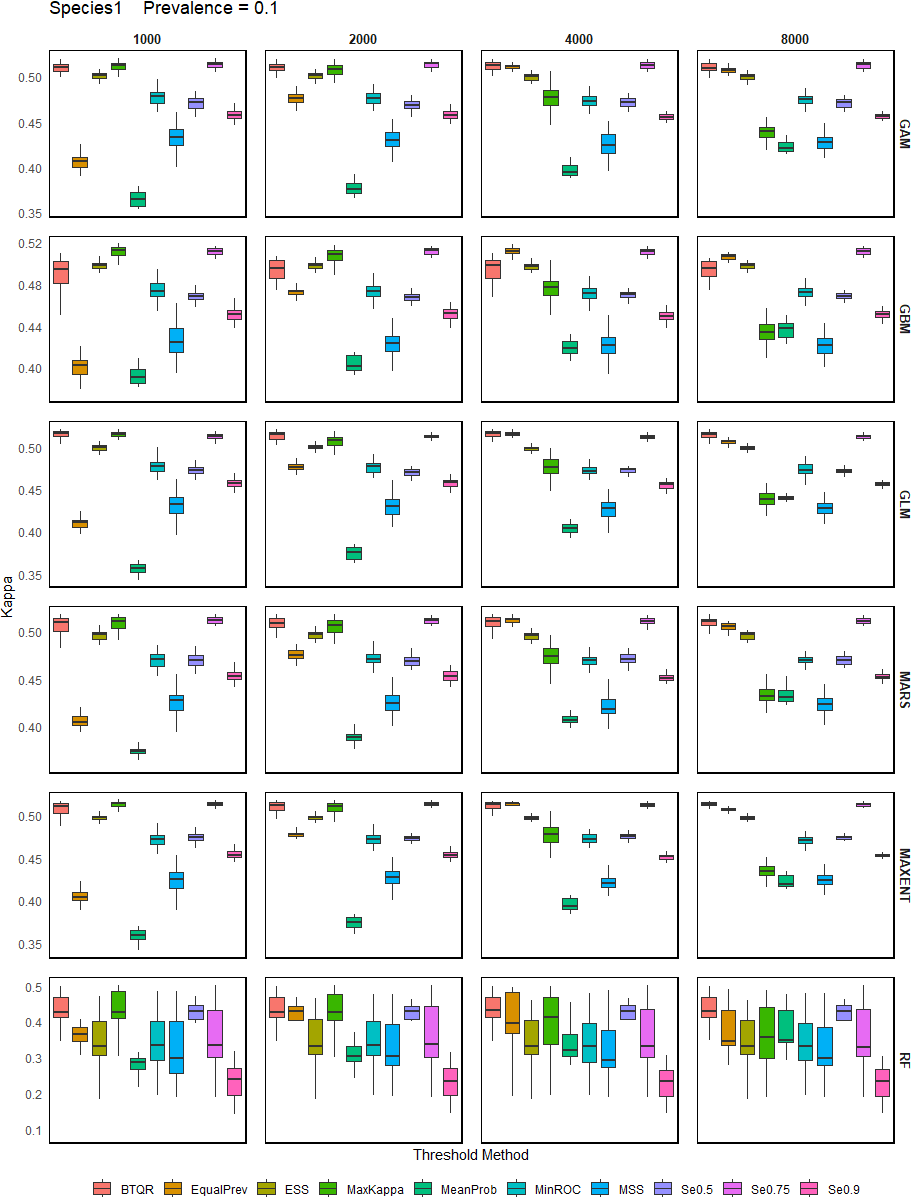


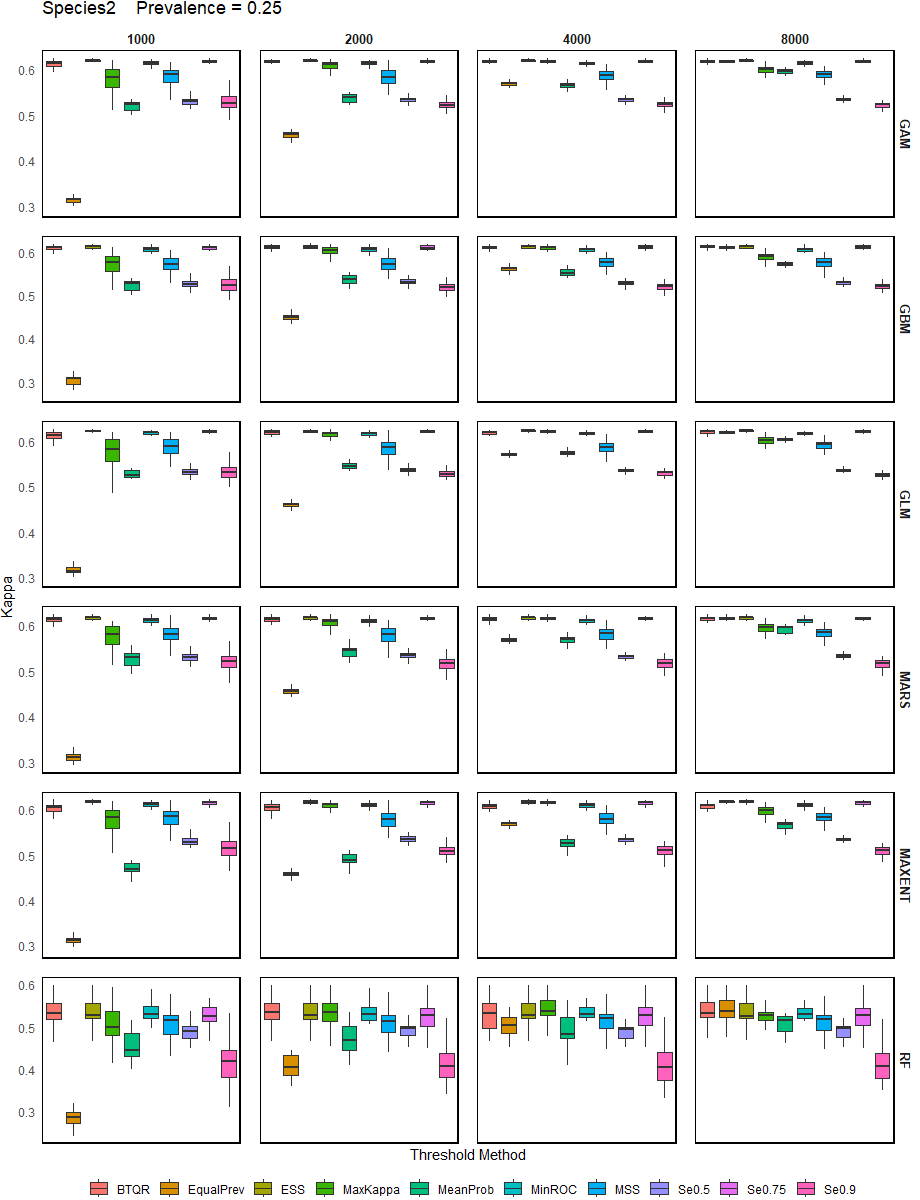


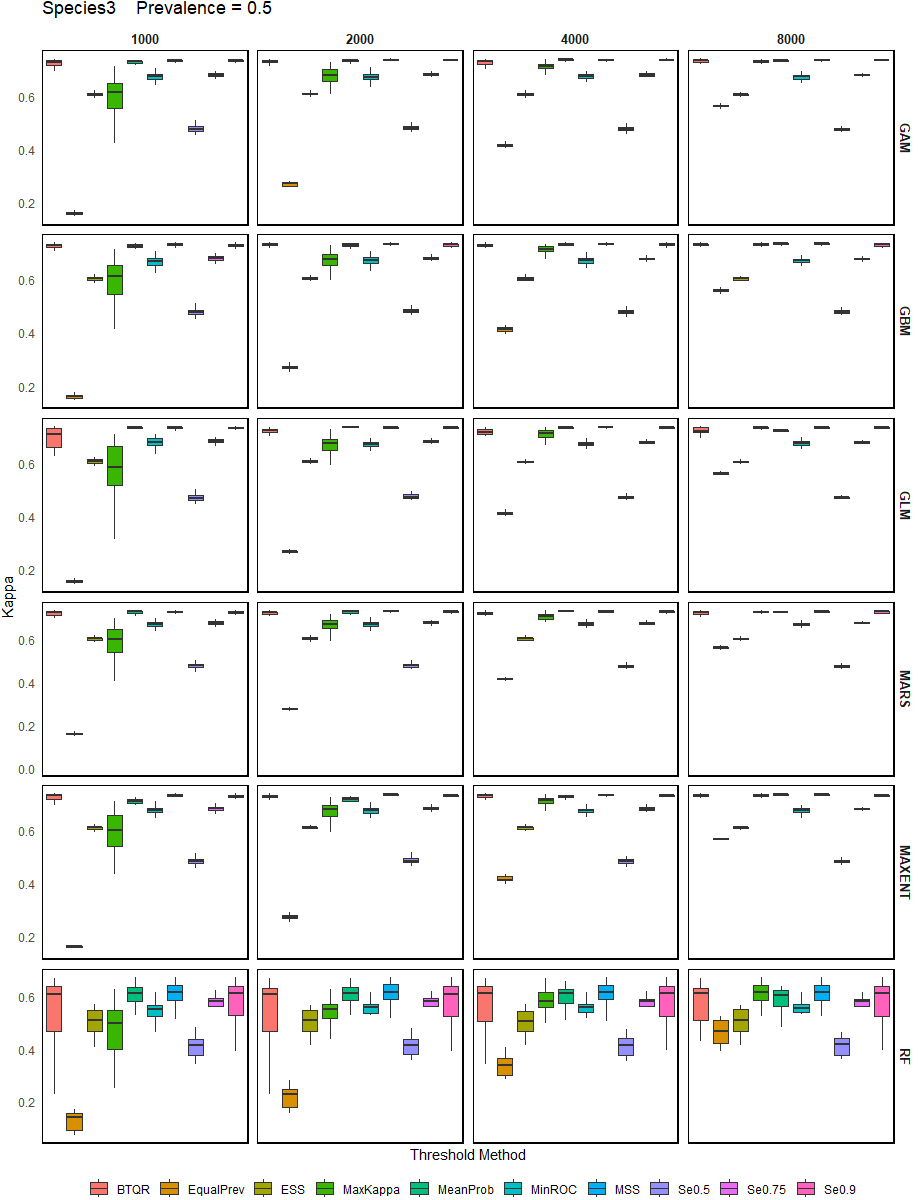


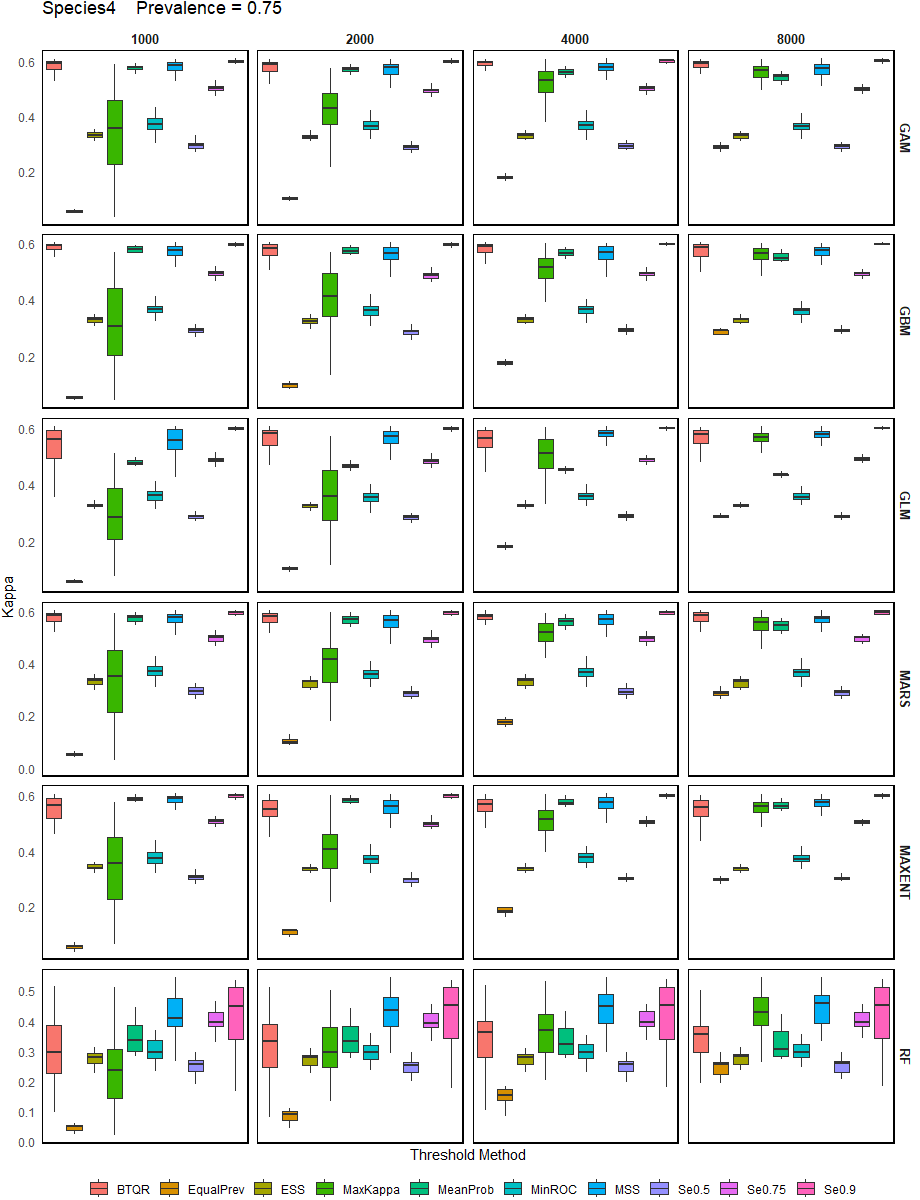

Supplement: Supplementary file 1 — Appendices S1–S3. [file ECE3-14-e11208-s001.docx]
